# Antigen-Specific versus Non-Antigen-Specific Immunoadsorption in ABO-Incompatible Renal Transplantation

**DOI:** 10.1371/journal.pone.0131465

**Published:** 2015-06-29

**Authors:** Gerold Thölking, Raphael Koch, Hermann Pavenstädt, Katharina Schuette-Nuetgen, Veit Busch, Heiner Wolters, Reinhard Kelsch, Stefan Reuter, Barbara Suwelack

**Affiliations:** 1 Department of Medicine D, Division of General Internal Medicine, Nephrology and Rheumatology, University Hospital of Münster, Münster, Germany; 2 Institute of Biostatistics and Clinical Research, University of Münster, Münster, Germany; 3 Department of General Surgery, University Hospital of Münster, Münster, Germany; 4 Institute of Transfusion Medicine and Transplantation Immunology, University Hospital of Münster, Münster, Germany; UNIFESP Federal University of São Paulo, BRAZIL

## Abstract

**Introduction:**

ABO-incompatible (ABOi) renal transplantation (RTx) from living donors is an established procedure to expand the donor pool for patients with end stage renal disease. Immunoadsorption (IA) is a standard procedure for the removal of preformed antibodies against the allograft. In this study, antigen-specific and non-antigen-specific IA in ABOi RTx were compared.

**Patients and Methods:**

10 patients underwent antigen-specific IA (Glycosorb group) and 13 patients non-antigen-specific IA (Immunosorba group). The effects of both procedures regarding antibody reduction, number of treatments, complications, costs, as well as the allograft function and patient survival were compared between both groups.

**Results:**

Although the IgG levels were reduced equally by both procedures (p=0.82), the reduction of the IgM level was more effective in the Glycosorb group (p=0.0172). Patients in both groups required a median number of 6 IA before ABOi RTx. Allograft function at one year after AB0i RTx was similar in both groups (estimated glomerular filtration rate: 66 vs. 64 ml/min/1.73m² respectively), with a death-censored graft survival of 90.0% and 92.3% respectively. Complication rates did not differ between procedures. Due to the reuse of non-antigen-specific Immunosorba columns, costs were considerably lower in this group; however, the use of the Immunosorba-based IA was less time-efficient.

**Conclusion:**

Considering upcoming alternatives as simultaneous performance of dialysis and IA or a possible reuse of Glycosorb columns, this might become less relevant in the future.

## Introduction

As the shortage of deceased donor kidney transplants is a growing problem for patients suffering from end stage renal disease (ESRD), ABO-incompatible (ABOi) living renal transplantation (RTx) has become an established alternative procedure [1]. ABOi RTx expands the donor pool and offers the opportunity to reduce time on the waiting list. Furthermore, ABOi RTx recipients often profit from the beneficial effects of reduced cold ischemia time related to living organ donation [2].

Since early studies of ABOi RTx in the 1980^s^, the perioperative management has continuously improved [3, 4]. Recently, excellent results of death-censored graft survival rates up to 100% after 24 and 36 months median follow-up period have been published [5, 6], and both graft and patient survival rates are now similar to those of ABO-compatible (ABOc) allograft recipients [7]. As induction and conventional immunosuppressive regimens are insufficient for the reduction of the ABO-isoagglutinin and human leucocyte antigen (HLA) antibody levels, desensitization therapies preventing blood-group antibody mediated rejection by means of intravenous immunoglobulins, plasmapheresis (PPh) and immunoadsorption (IA), or a combination of these procedures had to be established in protocols of ABOi RTx [8, 9]. Recently, Opelz et al. published data from a 3 year study suggesting that IA is superior to PPh-based protocols in ABOi recipients regarding allograft survival [7]. Most European desensitization protocols are based on the IA according to Tyden et al. [4]. This procedure involves an antigen-specific IA system (Glycosorb-ABO, Glycorex Transplantation, Lund, Sweden) utilizing single-use, low-molecular weight carbohydrate columns with immobilized blood-group A or B antigens linked to a sepharose matrix. These columns specifically deplete anti-A or anti-B antibodies and the use of the Glycosorb IA together with the application of anti-CD20 antibody rituximab showed excellent results after a 5 year follow-up [10].

Another IA principle is based on the parallel use of two regenerative columns containing protein A bound to a sepharose matrix (Immunosorba Fresenius Medical Care, Bad Homburg, Germany). Protein A binds the IgG subclasses 1, 2 and 4 with high affinity, and IgG3, IgA and IgM with lower or variable affinities without antigen specificity [11]. The depletion of non-antigen-specific antibodies might be advantageous when other, potentially harmful alloantibodies (e.g. HLA antibodies) need to be removed; however, other essential antibodies, e.g. those protecting the patients from infections, are also removed. Therefore, a comparison of both treatment strategies is of high interest to the transplant community. So far, only the non-antigen-specific IA using Therasorb columns has been compared with antigen-specific Glycosorb IA in a small study [12]. The aim of this study was to compare Immunosorba- and Glycosorb-based IA treatment strategies.

## Materials and Methods

### Study population and clinical data

Data were analyzed from patients who underwent ABOi RTx between November 2009 and March 2014 at the University Hospital of Münster, Germany. IA was started 7 days before the scheduled RTx date with a target-IgG level titer ≤1:8, and surgery was only performed when the titer was within the target range. During each IA 1.5 to 2 plasma volumes per patient were processed. From November 2009 to September 2012 the Glycosorb-ABO system was used in 10 patients (“Glycosorb group”), followed by the use of unspecific protein A column IA in 13 patients from October 2012 to March 2014 (“Immunosorba group”). In patients who were already on hemodialysis, IA was performed after hemodialysis.

Additional PPh with fresh frozen plasma was performed when Ig titers did not drop as expected. In all patients rituximab (Mabthera) was administered 30 days prior to the scheduled RTx (375 mg/m^2^ body surface area). Intravenous immunoglobulins (0.5 g/kg BW) were given once following the last preoperative IA treatment. Further immunosuppressive therapy was started 7 days before transplantation using tacrolimus (Tac, Prograf), mycophenolate mofetil (CellCept) and prednisolone (Soludecortin H / Decortin H). An induction therapy with basiliximab (Simulect) was given to all 23 patients at days 0 and 4. Tac was started at a dose of 0.1 mg/kg BW per day, with a target trough level of 8–10 ng/mL before ABOi RTx and during the first month, 6–10 ng/mL from month 2 to 3 and 3–8 ng/mL thereafter. Mycophenolate mofetil was given 1 g bid, and dosage was reduced in case of adverse events like leukopenia, diarrhea and infections. Prednisolone was started with 500 mg intravenously (i.v.) before RTx, 250 mg at day 1, followed by 100 mg for 3 days. It was then reduced by 20 mg/day. A dosage of 20 mg/day was maintained until day 30 and then slowly tapered to a final dosage of 5 mg/day after 6 months. Detailed information regarding the immunosuppressive regimen and trough levels is given in Table 1.

**Table 1:** Immunosuppressive regimen data.

	Glycosorb (n = 10)	Immunosorba (n = 13)	p value
Prednisolone dose			
at hospitalization	30	30	-
at RTx	500	500	-
1 week	20 (20–250)	20	0.849^a^
1 month	20 (15–20)	15 (10–20)	0.173^a^
3 months	10 (5–15)	7.5 (5–10)	0.122^a^
6 months	5 (5–7.5)	5 (5–7.5)	0.702^a^
12 months	5	5 (5–10)	-
Tacrolimus dose			
at hospitalization	7.4 ± 2.0	6.8 ± 1.1	0.403^b^
at RTx	8.5 ± 2.5	7.8 ± 1.5	0.426^b^
1 week	8.5 ± 2.0	8.0 ± 3.2	0.642^b^
1 month	8.1 ± 1.5	7.4 ± 2.6	0.475^b^
3 months	6.1 ± 2.1	5.1 ± 2.5	0.334^b^
6 months	5.1 ± 1.3	4.9 ± 2.6	0.787^b^
12 months	4.7 ± 1.7	4.1 ± 1.5	0.453^b^
Tacrolimus blood trough level			
at RTx	8.6 ± 2.5	9.8 ± 2.3	0.219^b^
1 week	8.2 ± 1.8	8.0 ± 1.8	0.844^b^
1 month	7.3 ± 1.3	7.8 ± 1.7	0.456^b^
3 months	7.6 ± 1.4	6.8 ± 1.9	0.286^b^
6 months	6.3 ± 1.3	5.9 ± 1.5	0.604^b^
12 months	5.8 ± 1.5	6.2 ± 1.3	0.594^b^
Mycophenolate mofetil dose			
at hospitalization	2000	2000	-
at RTx	2000	2000	-
1 week	2000 (1000–2000)	2000 (1000–2000)	0.226^a^
1 month	1000 (1000–2000)	2000 (1500–2000)	0.0704^a^
3 months	1250 (500–2000)	1000 (1000–2000)	0.576^a^
6 months	1000 (0–2000)	1000 (0–2000)	0.678^a^
12 months	1000 (0–1500)	1000 (0–2000)	0.354^a^

^a^ Mann-Whitney-U-Test;

^b^ t-test; same dose in all patients when no p value is provided.

All CMV positive patients received prophylactic valganciclovir for 100 days, D-/R- patients did not receive any prophylaxis, whereas D+/R- patients received prophylaxis for 200 days. Trimethoprim/sulphamethoxazole was given for 100 days after transplantation. The following data were collected from patient files: data of the donors and recipients, data of the IA and PPh, HLA mismatches of transplants, incidences of delayed graft function (DGF, defined as dialysis within the first week after RTx), blood types of donor and recipient, prior numbers of RTx, cold ischemia times (CIT) and warm ischemia times (WIT), CMV status before transplantation (donor and recipient), CMV infections (considered as relevant with ≥1.000 copies/μl), BK virus (BKV) infections (considered as relevant with ≥10.000 copies/μl blood). Histologic results were obtained from protocol biopsies at 3 months post transplantation and further indication biopsies following a decline in allograft function. All biopsies were reviewed by one pathologist according to the revised Banff criteria [13]. Data characterizing the allograft function, death and the cause of death of the patients were collected retrospectively. Data of all patients were anonymized prior to analysis. Written informed consent was given by all participants at the time of transplantation for recording their clinical data and collecting blood samples that were used in further analysis. The study was approved by the local ethics committee (Ethik Kommission der Ärtzekammer Westfalen-Lippe und der Medizinischen Fakultät der Westfälischen Wilhelms-Universität, No. 2014-390-f-N).

### Laboratory parameters

Patients showing HLA panel reactive antibodies (PRA) of ≥5% at the time of transplantation were defined as HLA-antibody-positive. PRA were measured by CDC against a panel of 56 T-lymphocytes (Lymphoscreen ABC 60, Bio-Rad Laboratories, Munich, Germany). Non-cytotoxic HLA antibodies were detected by ELISA (AbScreen HLA-Class I and II, Bio-Rad Laboratories, Munich, Germany). A Luminex bead array (LabScreen single antigen HLA-Class I and II, One Lambda, Canoga Park, CA, USA) was performed to specify HLA antibodies including donor-specific antibodies. HLA-specificities with mean fluorescence intensity (MFI) values >500 fluorescence units (FLU) were considered as positive. Based on the Luminex data, a virtual PRA was calculated using the Virtual PRA Calculator provided by the Eurotransplant Reference Laboratory (http://www.etrl.org). The Anti-IgG warm reactive (“IgG-titer”) and NaCl cold reactive titers (“IgM-titer”) of the ABO-bloodgroup isoagglutinins were assessed by incubation of the patient’s serum with stored red blood cells of the donor using the Diamed ID-Card gel card technology (Bio-Rad, Munich, Germany). The titer of serum isoagglutinins was determined using a two-fold serial dilution pattern of the serum in isotonic saline. 25μl of each serum dilution were incubated with 50μl of a 0.8% (v/v) red blood cell suspension of the donor in ID-Diluent 2 on the ID-Cards “Coombs-Anti-IgG” and “NaCl”, “Enzyme Test and Cold Agglutinins” and incubated at 37°C or room temperature for 15 min. The highest dilution of serum showing clear visible agglutination was designated as the isoagglutinin titer. Whole blood was analyzed for creatinine (enzymatic assay; Creatinine-Pap, Roche Diagnostics, Mannheim, Germany). The renal function was determined by eGFR calculation using the 4-variabel modification of diet in renal disease (MDRD) Study [14]: eGFR (ml/min per 1.73 m^2^) = 175 * serum creatinine^-1.154^ * age^-0.203^ * race * gender (race: 1.212 if black, otherwise 1; gender: 0.742 if female, otherwise 1).

### Statistical analyses

Statistical analyses of retrospective data were performed using SAS software, Version 9.4 for Windows and IBM SPSS Statistics 22 for Windows (IBM Corporation, Somers, NY, USA). Inferential statistics were intended to be exploratory (to generate hypotheses) instead of confirmatory, and were interpreted accordingly. Thus, p-values were interpreted in Fisher's sense, representing the metric weight of evidence against the respective null hypothesis of no effect. Neither a global significance level nor local levels were determined. P-values were considered noticeable if <0.05 and highly noticeable if ≤0.01.

All p-values are two-sided. Standard univariate statistical analyses were used to describe demographic and clinical parameters. Categorical variables are shown as absolute and relative frequencies.

Normal-distributed continuous variables are shown as “mean ± standard deviation” and were analyzed by T-tests. Non normal-distributed continuous variables are reported as “median [minimum - maximum]”. The Mann-Whitney U test was performed for comparison. Regarding boxplots, mild outliers are defined as values more than 1.5*interquartile range (IQR) from the rest of the scores and extreme outliers as values more than 3*IQR from the rest of the values.

This study includes only a small number of patients. Therefore, statistical analysis has to be interpreted only as a trend for different characteristics.

## Results

### Descriptive statistics

The 23 patients who underwent ABOi RTx were between 19 and 68 years of age (46.1±13.8) and 15 (65%) were male. Table 2 shows the characteristics of the patients of both groups and their corresponding donors. The patients in the Glycosorb group were approximately 10 years older than patients from the Immunosorba group (not statistically noticeable). The prolonged mean WIT in the Glycosorb group was caused by the reconstruction of the arteria renalis in two cases. Further characteristics did not differ significantly between the two groups.

**Table 2:** Patients`characteristics.

	Glycosorb (n = 10)	Immunosorba (n = 13)	p value
age (yr)	50.2± 12.9	39.3±14.0	0.088^a^
weight	78.9±21.2	71.6±11.4	0.305^a^
height	1.76±0.09	1.72±0.08	0.282^a^
BMI	25.2±4.7	24.3±3.3	0.579^a^
gender (m/f)	8 (80%) / 2 (20%)	7 (54%) / 6 (46%)	0.379^b^
reason for ESRD			
IgA nephropathy	3	2	-
ADPKD	2	2	-
minimal change GN	1	1	-
Alport-syndrome	1	-	-
chronic GN/unknown	3	3	-
interstitial nephritis	-	2	-
refluxnephropathy	-	1	-
FSGS	-	1	-
renal anti-GBM-syndrome	-	1	-
prior transplantation (yes/no)	3 (30%) / 7 (70%)	2 (15%) / 11 (85%)	0.618^b^
blood group (donor → recepient)			
A_1_ → O	4 (40%)	7 (54%)	-
A_2_ → O	2 (20%)	1 (8%)	-
B → O	2 (20%)	2 (15%)	-
A_1_B → O	1 (10%)	-	-
A_1_ → B	-	1 (8%)	-
A_2_ → B	1 (10%)	-	-
B → A	-	2 (15%)	-
preemptive transplantation	2 (20%)	4 (31%)	-
time on dialysis (months)	19.5 (0–69)	5.0 (0–23)	0.091^c^
number of HLA mismatches (0–6)	4.2±1.5	3.0±1.8	0.570^a^
PRA (≥5% CDC)	0	0	-
vPRA (≥5% Luminex)	3	2	-
DSA (HLA-specific)	2	2	-
CIT (hr)	2.55±0.97	2.07±0.43	0.127^a^
WIT (min)	37±24	22±12	0.063^a^
donor data			
donor age	54.3±9.1	54.1±6.6	0.946^a^
donor gender (m/f)	5 (50%) / 5 (50%)	6 (46%) / 7 (54%)	1^b^
genetic relation to recepient	3 (30%)	6 (46%)	0.669^b^
rituximab absolut (mg)	754±146	713±108	0.452^a^
rituximab application (days before RTx)	30.5±6.9	14.5±17.8	0.716^a^
postoperative hospital stay	25.5 (17–56)	19.0 (13–44)	0.067^c^

^a^ t-test;

^b^ Chi2-test;

^c^ Mann-Whitney-U-Test. All patients were Western European descent. ESRD, end stage renal disease; BMI, body mass index; ADPKD, autosomal dominant polycystic kidney disease; FSGS, focal segmental glomerulosclerosis; GN, glomerulonephritis; GBM, glomerular basement membrane; HLA, human leukocyte antigen; PRA, panel reactive antibodies, vPRA, virtual panel reactive antibodies; CIT, cold ischemia time; WIT, warm ischemia time.

### IA and PPh data

Table 3 shows the median and absolute numbers of IA and PPh treatments and the resulting IgM—and IgG-antibody titers. Patients of both IA groups required similar numbers of IA treatments before and after transplantation. Two patients of both groups had HLA-specific DSA before ABOi RTx. Only one patient of the Immunosorba group required 30 IA sessions due to the presence of strong HLA-DQ-antibodies. This patient experienced an acute antibody-mediated rejection (ABMR) episode and a T cell-mediated rejection (TCMR) 5 months after transplantation. After successful treatment (prednisolone and PPh) the eGFR was 52 and 61 ml/min/1.73m^2^ 6 and 12 months after RTx, respectively.

**Table 3:** Immunoadsorption (IA).

	Glycosorb (n = 10)	Immunosorba (n = 13)	p value
preoperative IA	6 (2–17)	6 (4–30)	0.472^a^
patients needed postoperative IA	3 (30%)	2 (15%)	-
number of postoperative IA	0 (0–8)	0 (0–10)	0.548^a^
mean IA duration (min)	187±38	186±34	0.933^b^
processed IA plasma volume (ml/kg BW)	74.0±8.0	80.1±12.3	0.187^b^
IgG level before IA	1:32 (1:4–1:2048)	1:32 (1:8–1:512)	0.248^a^
IgG level before RTx	1:4 (0–1:8)	1:4 (0–1:8)	0.825^a^
IgG level 1 week after RTx	1:2 (0–1:8)	1:4 (0–1:8)	0.570^a^
IgG level 2 weeks after RTx	1:2 (0–1:16)	1:2 (0–1:8)	0.614^a^
Ig M level before IA	1:16 (1:4–1:128)	1:8 (1:1–1:32)	0.413^a^
IgM before RTx	0 (0–1:2)	0 (0–1:2)	-
IgM level 1 week after RTx	0 (0–1:4)	0 (0–1:8)	0.909^a^
IgM level 2 weeks after RTx	0.5 (0–1:8)	0 (0–1:16)	0.890^a^
processed PPh plasma volume (ml/kg BW)	30.9 (17.2–61.9)	49.3 (19.0–89.8)	0.0586^a^
patients who underwent additional PPh before RTx	6 (60%)	10 (77%)	0.65^c^
number of PPh before RTx	1 (0–8)	2 (0–5)	0.824^a^
patients needed additional PPh after RTx	2 (20%)	2 (15%)	-
number of PPh after RTx	0 (0–16)	0 (0–5)	0.673^a^
platelet count before IA	220±70^d^	232±63^e^	0.0488^d^
platelet count after IA	166±73^d^	169±54^e^	0.001^e^

^a^ Mann-Whitney-U test;

^b^ t-test;

^c^ Chi^2^ test;

^d^ paired t-test of the platelet count before and after IA in the Glycosorb group;

^e^ paired t-test of the platelet count in the Immunosorba group; PPh, plasmapheresis; RTx, renal transplantation; BW, bodyweight.

**Fig 1:**
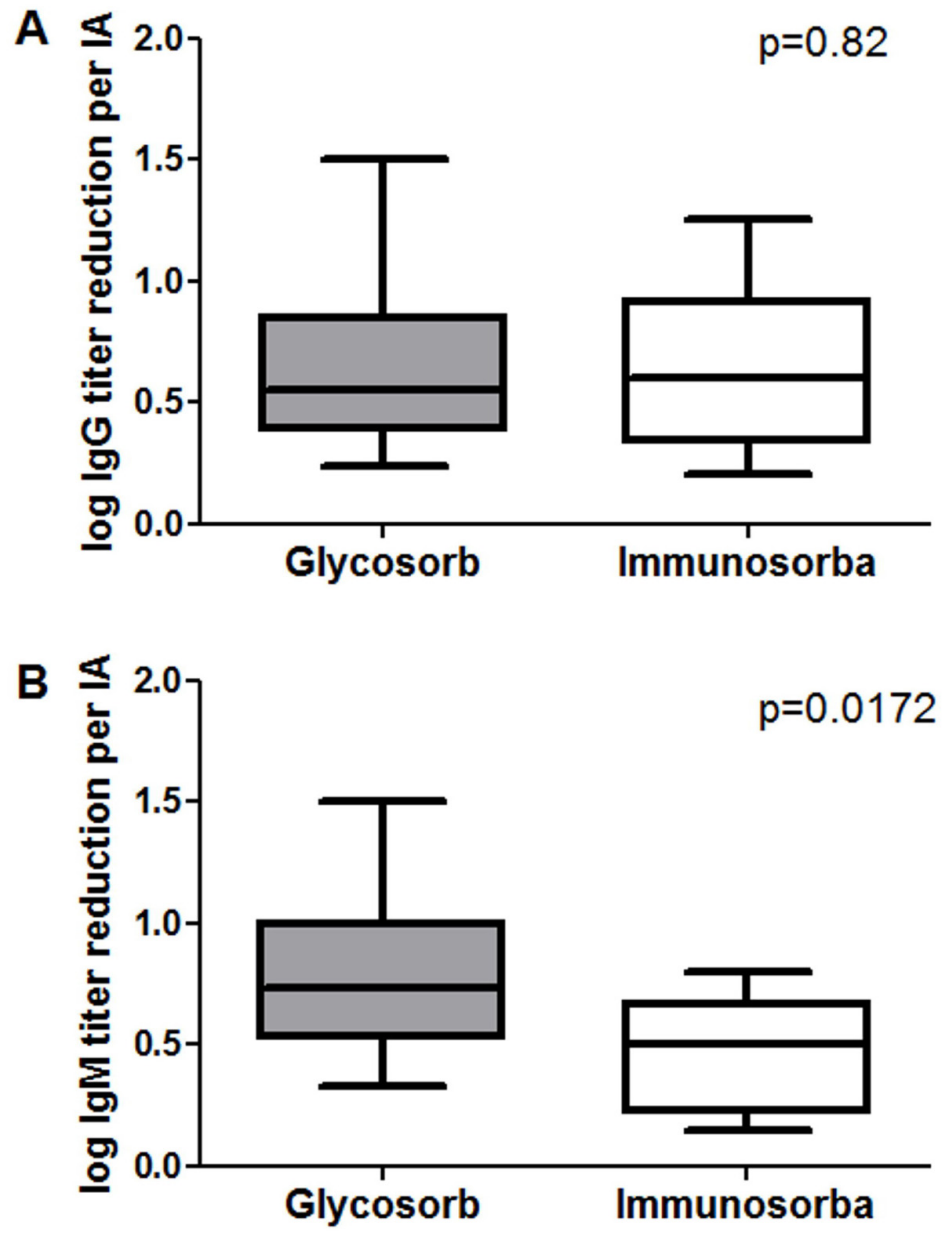
Reduction of Ig levels. (A) The reduction of IgG level per IA (shown in logarithmic steps) showed no noticeable differences in terms of efficacy between the IA groups (0.55 (0.24–1.5) vs. 0.6 (0.2–1.25), p = 0.82) (B) IgM levels were reduced more effectively by the antigen-specific Glycosorb IA (0.73 (0.33–1.5) vs. 0.5 (0.14–0.80), p = 0.0172).

**Fig 2:**
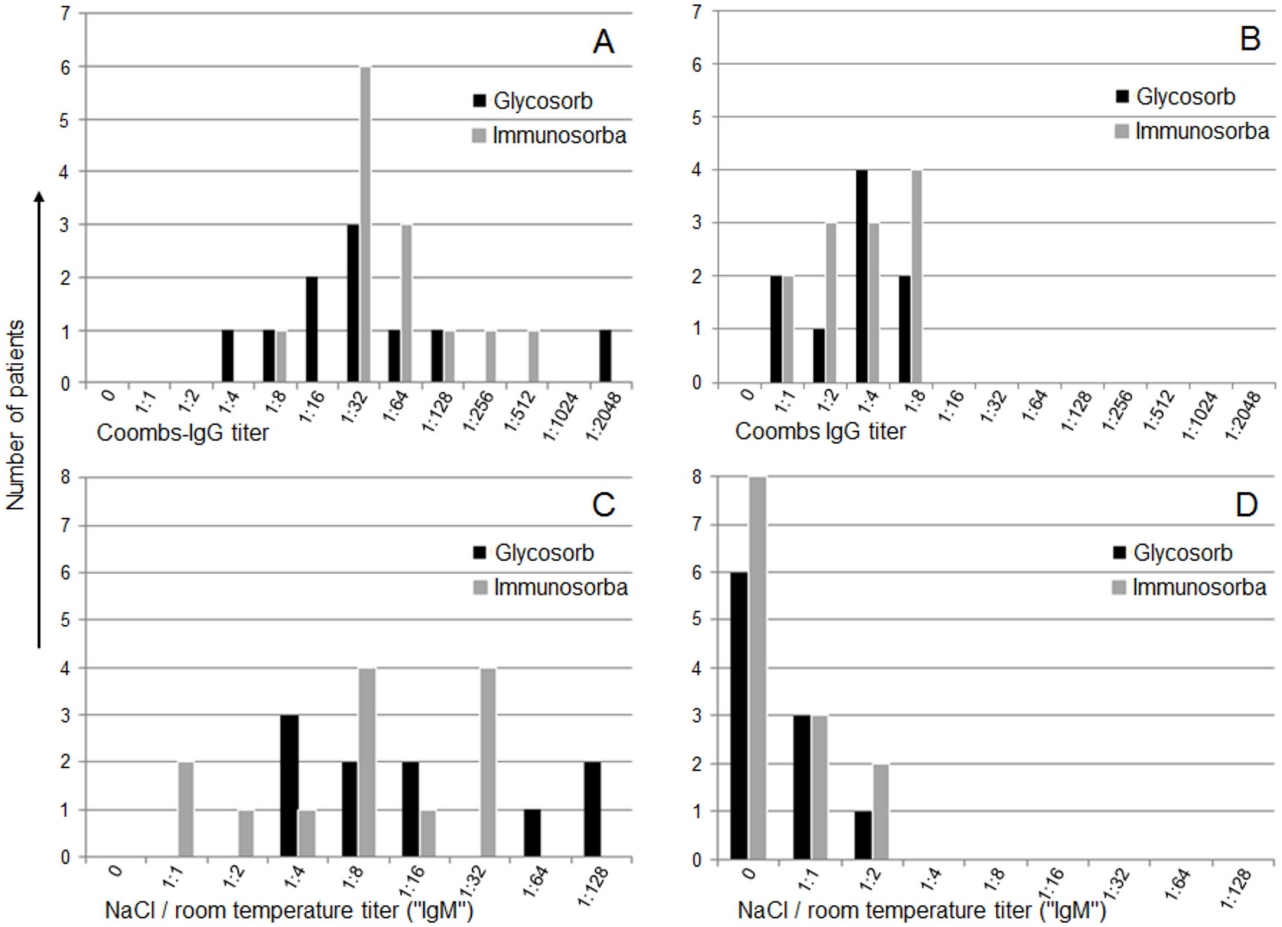
IgG- and IgM-titers (log steps) for each patient before first IA and before ABOi RTx. (A) IgG titers before first IA. (B) IgG titers before RTx. (C) IgM titers before first IA. (D) IgM titers before RTx.

Although additional PPh before ABOi RTx were performed in 60% of the Glycosorb group and in 77% of the Immunosorba patients, the number of PPh treatments in each patient before ABOi RTx was low. Postoperative IA and PPh until the first discharge from our hospital were rarely required. The efficiency of IgG antibody reduction—related to the number of performed IA—was not different between the study groups (Fig 1, p = 0.82). IgM titer reduction was noticeable more effective in the Glycosorb group, when compared to the non-antigen-specific IA (Fig 1, p = 0.0172). The IgG- and IgM-titers for each patient before the first IA and before ABOi RTx are shown in Fig 2. Platelet counts during the IA treatments were decreased in both groups (Table 3). Costs for Glycosorb IA were 3750 € for each column including the IA system, multiplied by the total number of further IA sessions, since Glycosorb columns are not approved for reuse (Table 4). The Immunosorba columns (2700 € per column) were generally reused for all IA sessions for the same patient. Thus, the overall costs for this treatment were considerably lower compared to the antigen-specific IA Glycosorb system (Table 4).

**Table 4:** Costs for immunoadsorption.

	Glycosorb	Immunosorba
IA column incl. disposables 19% tax	3800	2700
6 treatments (median)	22800	6150
all 69 treatments prior to RTx	262200	-
all 117 treatments prior to RTx	-	106860
all 11 treatments after RTx (3 patients)	41800	-
all 15 treatments after RTX (2 patients)	-	10320

All prices are shown in Euro. Expenses do not include replacement fluids and labor costs.

### Renal function

The death-censored graft survival 12 months post RTx was similar in both groups (90.0% in the Glycosorb group vs. 92.3% in the Immunosorba group). One patient of the Glycosorb group experienced renal graft loss 107 days after ABOi RTx due to a severe ABMR.

A patient of the Immunosorba group suffered from a haemolytic uremic syndrome (HUS) a few days after RTx, which was primarily thought to be tacrolimus-induced. Hence, PPh was started, but due to severe allergic reactions during the first procedure, it was switched to IA (10 sessions) before eculizumab was given. Furthermore, the immunosuppressive therapy was switched from tacrolimus to everolimus. 6.6 months after RTx this patient underwent allograft loss due to HUS, followed by several humoral and cellular allograft rejections and recurring pulmonary infections and UTI. The patient died from a bradycardia during hemodialysis 13.3 months after transplantation.

**Fig 3:**
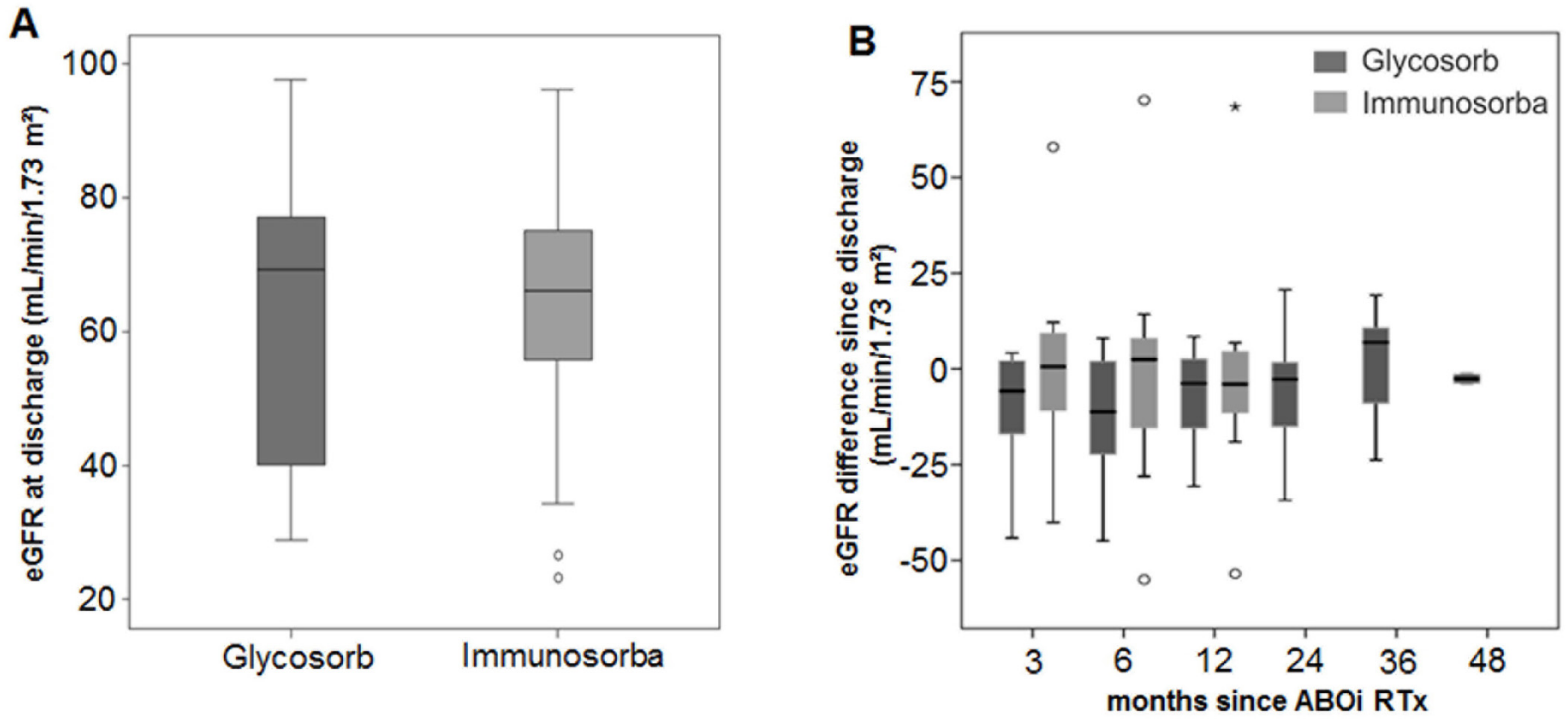
Comparison of renal function in both procedures. (A) The renal function (eGFR values calculated by MDRD) of patients who underwent antigen-specific and non-antigen-specific IA did not differ when patients were discharged from our hospital (p = 0.643). (B) During a 12 months follow-up, the eGFR of both IA groups revealed no noticeable differences from the baseline eGFR or between the IA groups (p = 0.185). °mild outliers; *extreme outliers.

Overall renal function did not differ between the two IA groups at the first discharge from our hospital after ABOi RTx (Fig 3A) and in a 12 months follow-up (Table 5, Fig 3B).

**Table 5:** Renal function (eGFR in mL/min/1.73m^2^).

time after RTx	group	N	mean±SD	10% q.	25% q.	median (min-max)	75% q.	90% q.	skewness
at discharge	Glycosorb	10	64±23	30	40	69 (29–98)	77	91	-0.45
	Immunosorba	13	62±23	27	56	66 (23–96)	75	88	-0.49
3 months	Glycosorb	9	57±15	31	51	55 (31–84)	62	84	0.15
	Immunosorba	13	64±20	34	56	62 (32–96)	76	87	-0.18
6 months	Glycosorb	9	57±14	35	52	55 (35–73)	69	73	-0.20
	Immunosorba	13	61±23	36	49	58 (19–100)	76	97	0.08
12 months	Glycosorb	9	60±14	37	53	66 (37–73)	69	73	-1.00
	Immunosorba	11	66±20	47	61	64 (20–95)	78	88	-0.93
24 months	Glycosorb	9	62±16	41	49	69 (41–92)	69	92	0.26
36 months	Glycosorb	7	63±15	47	48	70 (47–80)	74	80	-0.20
48 months	Glycosorb	2	56±24	39	39	56 (39–73)	73	73	-

All eGFR values were estimated by the CKD-EPI formular, SD, standart deviation; q., quantil; only patients of the Glycosorb group reached the time point 24 months until the time of data analysis.

### Biopsy results

In 70% (Glycosorb group) and respective 77% (Immunosorba group) of the patients, at least one allograft biopsy was performed (Table 6). In 3 patients with stable renal function, a protocol biopsy was not performed due to concomitant anticoagulation therapy.

**Table 6:** Biopsy results.

	Glycosorb (n = 10)	Immunosorba (n = 13)	p value
biopsy performed	7 (70%)	10 (77%)	1^a^
first biopsy (days after RTX)	49.9±41.1	42.8±38.0	0.720^b^
observed rejecton	4 (40%)	4 (31%)	-
ABMR	2 (20%)	3 (23%)	-
ABMR due to HLA-specific DSA	-	2 (15%)	-
TCMR	2 (20%)	1 (8%)	-
borderline rejection	3 (30%)	3 (23%)	-
BK nephropathy	0	1 (8%)	-
CNI nephrotoxicity	0	2 (15%)	-
thrombotic microangiopathy	0	1 (8%)	-

^a^ Chi^2^-test;

^b^ t-test; ABMR, antibody-mediated rejection; HLA, human leucocyte antigen; DSA, donor specific antibodies; TCMR, T cell-mediated rejection; CNI calcineurin inhibitor

Both groups showed similar incidence of biopsy-proven acute rejections. Two patients of the Immunosorba group who experienced an ABMR had HLA specific DSA (14 and 504 days after RTx). Nevertheless, all patients were successfully treated, either with PPh and prednisolone in case of ABMR or with prednisolone only in case of TCMR and borderline rejection. BK nephropathy and CNI nephrotoxicity occurred only in a small number of cases in the Immunosorba group.

### Complications

Typical surgical and infectious complications were observed in both groups (Table 7). The most severe surgical complication was a hematoma next to the transplant kidney, which occurred during the first hospital stay. Urinary tract infections (UTI) were the most common infections in both groups. CMV infection was observed in two patients of the Glycosorb group with both patients developing a slight increase in creatinine (447 respective 161 days after RTx), which returned to baseline after treatment with ganciclovir. One patient of the Immunosorba group experienced viral stomatitis 68 days after RTx. A severe fungal pneumonia occurred in one patient per group. These patients recovered completely after appropriate therapy. One patient of each group suffered from a combined bacterial and fungal pneumonia. The patient from the Glycosorb group experienced the pneumonia 769 days after RTx and died from a severe sepsis; aspergillus fumigatus and pseudomonas aeruginosa were found in the bronchoalveolar lavage and the graft was lost as a result of the multiple organ failure. The corresponding patient of the Immunosorba group recovered completely after treatment with piperacillin/tazocatam and fluconazole. The findings of the CT chest scan were highly suggestive of fungal pneumonia, but the causing agent could not be identified.

**Table 7:** Complications.

	Glycosorb (n = 10)	Immunosorba (n = 13)	p value
DGF	1	1	-
sugical complications			
wound infection	3	1	-
Hematoma	4 (40%)	5 (38%)	1
Lymphocele	3	3	-
urinary leakage	1	0	-
dissection of renal artery	1	0	-
Infections			
BK viremia	0	1	-
CMV disease	2	0	-
urinary tract infections	5 (50%)	5 (38%)	0.685
bacterial pneumonia	1	2	-
combined fungal pneumonia	1	1	-
bacterial meningitis	1	-	-
Stomatitis	-	1	-
12 months graft loss	1 (10%)	1 (7.7%)	0.846
Death	1	1	-

Chi^2^-test; DGF, delayed graft function; CMV, cytomegalovirus

## Discussion

Desensitization procedures using antigen-specific IA in ABOi kidney transplant recipients lead to considerably good graft and patient survival rates. However, non-antigen-specific IA with reusable Protein A columns has emerged as an attractive alternative [12]. Morath et al. already concluded from a pilot study conducted with Therasorb or Glycosorb columns that reusable non-antigen-specific IA devices are more cost-effective than antigen-specific IA devices for the depletion of potential human leukocyte antigen-alloantibodies [12]. Considering the recently described “off-label” re-use of the antigen-specific IA column this difference might become less important in the future [15].

Herein, for proof-of-concept, the non-antigen-specific Immunosorba column was compared to the antigen-specific Glycosorb column for desensitization of ABOi RTx recipients.

We used a modified Stockholm protocol for desensitization of recipients. Patients with IgG isoagglutinin titers above 1:128 (Coombs technique, range 1:4–1:2048) were included in our study. However, the median isoagglutinin titer in both groups was low (1:32) but comparable to data presented by Morath et al. [12]. The maximum acceptable isoagglutinin titer, before a transplantation was performed, was set to ≤1:8 (according to the Stockholm protocol) [16]. This is a more restrictive strategy when compared to other transplantation centers, accepting 1:32 or 1:16 as target titers [12]. Due to the limited experience with ABOi RTx at the University Hospital of Münster, several IA in 3 patients revealing IgG levels ≤1:8 before RTx were also performed, although in a recently published study, Masterson et al. demonstrated that antibody removal is not necessary in patients with low anti-blood group antibodies [17]. In the present study, postoperative IA treatments were only performed when the titer increased above 1:8 and a significant rise in creatinine (> 0.3 mg/dL) occurred within the first 2 weeks. According to Morath and other investigators, the daily routine administration of intravenous immunoglobulins was not conducted, but basiliximab induction therapy was introduced into the protocol for all ABOi RTx patients [7, 12].

### Desensitization

Successful desensitization in our cohort was independent of the IA column used. Morath et al. who failed to achieve a sufficient reduction in isoagglutinin titers by solely use of IA in one third of patients supposed that the observed treatment failure with IA, and the beneficial effects of additional PPh, might be explained by the presence of larger amounts of isoagglutinins of the IgG3 or IgM isotype, which can be removed more easilyby PPh than by IA [12]. It has been reported that in cases of insufficient titer reduction by IA, PPh might be a therapeutical option, although the IgG subtype was not investigated [18]. For this reason, we performed additional PPh between IA sessions when the drop in IgG titers was considered insufficient. This was necessary in 60% of patients in the Glycosorb group (usually one additional PPh per patient) and in 77% of patients in the Immunosorba group (usually two additional PPh per patient) in our study. The drop of the IgG titer during each session was quite similar in both groups and desensitization was successfully performed in all patients. Additionally, our data show that the reduction of IgM is at least as good as the IgG reduction, and the median IgM titer was zero for patients at the time of transplantation. Interestingly, in the Glycosorb group, the IgM titer was reduced more effectively with each IA session; however, the clinical impact of this potential advantage is unknown. The total plasma volumes processed tended to be higher in the Immunosorba group (5.2 L vs. 8.2 L). This is in accordance with observations by Morath et al. [12], revealing that a plasma volume of 6.4 L per session had to be treated during antigen-specific IA compared to 7.9 L during non-antigen-specific IA. It might be possible that these concurrent findings are only random. Notably, in the study of Morath et al. the median baseline isoagglutinin titers were 1:32 (Glycosorb) vs. 1:64 (Therasorb) while we assessed a median of 1:32 in both groups. The absolute number of sessions per patient was similar in both groups. In contrast Wahrmann et al. observed a better isoagglutinin clearance with all non-antigen-specific IA columns (Therasorb, Immunosorba and Globaffin) compared to the antigen-specific IA (Glycosorb) in a single IA session as well as in series of four sessions [19]. Interestingly, this was true for anti A/B IgG but not for IgM type isoagglutinins. However, during treament with non-antigen-specific columns significantly higher plasma volumes had to be processed. Moreover, in this study non-antigen-specific IA was applied outside the context of ABOi RTx. Baseline titers were very low (IgG 1:4) and less accurate gel card tests were used for titer assessment.

According to our data and to other studies, IgG titer reduction in Immunosorba-based protocols can be achieved as efficient as in Gylcosorb-based treatment, if the plasma volume per session is increased [12, 19]. This can be done with minimal effort, but is more time-consuming and thus might be critical in cases when additional regular dialysis is required or when the schedule is restricted. Alternative strategies, including simultaneous dialysis and IA treatment, might overcome this issue in the future [20].

### Complications

Non-antigen-specific IA could be of advantage in cases when other, potentially harmful HLA alloantibodies might play a role. In the present study, both procedures depleted HLA-specific DSA in two patients. However, one patient of the Immunosorba group developed an ABMR and TCMR, due to the existence of strong DSA. The overall rate of observed acute rejections in our cohort (40 and 31%) appears to be high, but is comparable to that in other studies [1, 6, 12]. Rejection episodes might be instigated following the reduction of immunosuppression (especially MMF) after previous infections. Another explanation might be the performance of protocol biopsies, as up to 50% of rejection episodes are subclinical only and not detected in studies in which only indication biopsies were conducted. [21, 22]. Particularly the relevance of borderline rejections detected by protocol biopsy without a significant rise in creatinine remains questionable [23].

Since protective antibodies might also be depleted by both IA procedures, we assessed the infection rate after RTx. It was hypothesized that the intensified (pre-) treatment of patients promotes infections and possibly infection-related cancer like Karposi sarcoma after RTx. Indeed, treatment with rituximab or splenectomy was reported to increase the risk for viral infections [24, 25]; however, Kahwaij et al. could not confirm these findings in patients following PPh and IVIG-based with or without rituximab desensitization [26]. In addition, Lentine et al. did not find significant more infections after splenectomy in ABOi RTx when compared to ABOi without splenectomy [27]. Recent data on infections after ABOi-RTx are inconsistent. Comparing ABOi RTx versus ABOc RTx using PPh without rituximab, Flint et al. found no significant differences in infections rates [28]. Using antigen-specific IA for desensitization, Habicht et al. observed a significantly higher overall infection rate in ABOi patients when compared to ABOc transplantation [29]. Viral infections (mainly BKV, CMV, HSV and VZV) tended to be more frequent, but this difference did not reach statistical significance. As the post surveillance calcineurin inhibitor levels as well as the doses of tacrolimus and MMF did not differ between the ABOi and ABOc group, they attributed their findings to the total burden of immunosuppression. In the present study BKV and CMV in the peripheral blood or in the biopsies were rarely positive and did not differ between both groups. The largest investigation and literature review analyzing post-transplant infections in ABOi RTx did not focus on viral infections, but revealed that ABOi RTx increases the risk for bacterial infections [27]. Notably, in this study, patients from the U.S. Renal Data System were investigated, a cohortin which mainly PPh-based desensitization is performed. In the largest European analysis, Opelz et al. followed 1420 ABOi RTx patients for three years and noted a slight increase of the infection rate in ABOi RTx, but only in the very early posttransplant period [7]. Overall, infection rate, type of infection, hospitalization and death did not differ between ABOi RTx and ABOc RTx. In accordance with our data, the most common infection in ABOi RTx was UTI. UTI was observed in up to 50% of our patients which is similar to published data from others [7, 27]. In summary, present data on infections are inconclusive, due to different treatment modalities and post surveillance protocols used in the various studies. In accordance with Morath et al., we were unable to detect any influence of the type of IA column on the infection rate in ABOi RTx.

Due to the depletion of coagulation factors, PPh is thought to cause more bleeding complications compared to IA. However, the development of hematomas or bleeding episodes after ABOi RTx is rarely reported by centers using PPh or double filtration plasmapheresis and prevalence seems to be low with <5% [8, 28, 30]. Several studies showed that the overall surgical complication rate was higher in ABOi RTx patients than in ABOc RTx patients [27, 31], with hematomas being the most frequently observed complication. In our study, surgical complications were not correlated with the use of a specific IA column which is in accordance to the data from Morath at al. [12]. It was hyothesized that bleeding complications in ABOi RTx are related to PPh and IA, which might cause consumption of platelets and coagulation factors as well as platelet dysfunction [32, 33]. Indeed, patients of both IA groups in our study showed a considerable decline of platelets during IA. In contrast, Renner at al. attributed the bleeding in 4 of 14 ABOi RTx patients to the amount of heparin used for the perfusion of the graft [34]. The use of heparin (1000 I.U./h) gives also a further explanation for the common development of hematomas post transplantation in our cohort. As a consequence of this observation, the heparin dose was reduced. De Weerd et al. could show that the absolute number of IA procedures performed correlated with the need for blood transfusions [35]. Thus, this effect seems to be generally related to IA and not to a specific column.

This study is somehow limited by the mainly retrospective analysis and short follow-up period. As the entire study population consists of Western Europeans, the data is only representative for this ethnical group with similar genetic characteristics. Although the study includes only a relatively small number of patients, this is the largest report comparing these modalities to date.

## Conclusions

We were able to effectively reduce isoagglutinin titers in all ABOi RTx patients regardless of the column used. Allograft function and complication rate were comparable between the antigen-specific and non-antigen-specific IA group. While Immunosorba-based IA was more cost- effective than Glycosorb, it was more time-consuming. However, as IA could be performed combined with dialysis or a reuse of Glycosorb columns might be performed in the future, these aspects might become less important.
